# Obstetrics

**Published:** 1904-06

**Authors:** W. C. Swayne


### OBSTETRICS.

In 1896 there appeared in this *Journal* an account of the then recent work on "**Deciduoma Malignum, (or Chorion-epithelioma,)**" with a statement as to the views held of its nature and origin.

A noticeable feature of the opinions current among British pathologists was that they differed in a very material degree from those of continental observers, the general tendency of British opinion being that this affection was to be considered as a condition in which sarcomatous or carcinomatous changes arose in the uterus in connection with, but not necessarily as, the result of conception and parturition. A noticeable feature of the previous communication was that the amount of material, as evidenced by cases reported in the United Kingdom, was very small as compared with that in the hands of continental observers.

Since the date of the last communication to this *Journal* the opinion of British observers has undergone a marked change in the direction of that previously held by continental observers, and, in addition to this, not only have a fair number of cases been reported, but the re-examination of museum specimens, described as malignant disease or malignant ulceration of the corpus uteri, has resulted in the discovery of instances of this affection which had up to the present passed unrecognised.

There can be no question that a number of cases of this affection have been classified as instances of malignant disease of the uterus without more special differentiation, and there can be no doubt that the increase in the number of cases observed has been largely due to the increased vigilance maintained over suspected cases and the more careful microscopical examination of fragments removed, by curette or otherwise, in cases in which the symptoms led to a suspicion that this affection might be a cause of the symptoms presented by the patient.

There are several striking features among some of the more recently reported cases of this affection. Russell Andrews[1] reports a case in which the disease arose eleven years after the last pregnancy. McCann[2] reports one nine years after the last pregnancy. Schmauch[3] reports a case in which, although syncytial metastases were found in the vagina, kidneys, lungs, spleen and brain, no primary growth was found in the uterus. Malcolm and Bell[4] report a case in which deposits of chorion-epithelioma were found in the uterus three weeks after the removal of a hydatid mole. Schlangenhaufer[5] reports two cases occurring in males, in one of which the patient died from "sarcoma of testicle with secondary deposits in lung, thyroid gland and kidney," and on minute examination the tumours were found to have the structure of chorion-epithelioma, while in another not only were cells found which corresponded to the epithelial cells of the chorionic villi, but mesoblastic tissues as well as, in fact, whole villi in a state of hydatidiform degeneration. These two cases, moreover, are by no means the only ones that have come to light. Risel[6] also derives these chorion-epitheliomatous proliferations from fœtal ectoderm, and considers them equivalent to the other ectodermal structures of teratomata. Pick,[7] while not denying the possibility that chorion-epitheliomata can arise from fœtal ectodermic material displaced in the system of the patient, which mode of origin he considers Risel to hold, is of opinion that they arise almost invariably from chorionic epithelium, the product of conception, as originally stated by Marchand. Ritchie[8] showed a case of embryona removed from the anterior mediastinum of a young man with metastases in the lungs, liver and spleen. Part of the tumour proved to be a dermoid cyst, and part to have the structure of chorion-epithelioma, as also had the metastases. F.W. Andrewes[9] at the same meeting showed two sections from specimens of endosteal sarcoma of the femur, in both of which syncytial masses were present.

From the foregoing the following points are to be noticed:—

[1] *Tr. Obst. Soc. Lond.*, 1903, xlv. 238. [2] *Ibid.*, 1902, xliv. 294.

[3] *Ztschr. f. Geburtsh. u. Gynäk.*, Bd. xlix., Hft. 3; abstract in *Brit. Gynæc. J.*, 1903, xix. summary, 138.

[4] *Tr. Obst. Soc. Lond.*, 1903, xlv. 483.

[5] *Wien. klin. Wchnschr.*, 1902, xv. 571, 604; quoted in *Tr. Obst. Soc. Lond.*, 1903, xlv. 287, 288.

[6] *München. med. Wchnschr.*, 1903, l. 1688; abstract in *Brit. Gynæc. J.*, 1903, xix. summary, 137.

[7] *Zentralbl. f. Gynäk.*, 1903, xxvii. 1110.

[8] *Tr. Obst. Soc. Lond.*, 1903, xlv. 250. [9] *Ibid.*, 237.

(1) That syncytial cell masses may occur in ordinary sarcoma

(2) That chorion-epithelioma growth containing syncytia masses and also proliferating epithelial cells may occur (*a*) in males, (*b*) in virgins.

(3) That the connection as regards time between the last pregnancy and the occurrence of the tumour is most variable, cases having been reported when the interval was as short as three weeks and as long as eleven years. Fleischmann[1] reports a case in which a chorion-epithelioma was removed from the vagina some months after the delivery of a hydatid mole, and also a nodule from the right uterine wall. Both these tumours consisted of a typical chorion-epithelioma. The patient recovered and remained well.

The most exhaustive paper that has appeared in British literature on this subject is one by Teacher.[2] He refers to the view taken by the members of the Obstetrical Society of London in 1896—that these tumours were only sarcomata of the uterus having an accidental association with pregnancy—as being the great stumbling block to the progress of the knowledge of this affection, and he presented three new cases in order to provoke a new discussion of the subject.

He defines chorion-epithelioma as follows:—"A malignant tumour developing in the body of the uterus about the time of a confinement or abortion," of which the clinical signs are the occurrence of severe hemorrhages, progressive anæmia, and cachexia, and resulting, if left to itself, in death in a period varying from weeks to months. Hemorrhagic growths are found in the uterus, and metastatic growths, also of a hemorrhagic character, in the vaginal walls and lungs.

Histologically it consists of masses of well-defined cells of various shapes and sizes closely packed together and large multi-nuclear irregular masses of protoplasm, in which no definite cell boundaries are recognisable. This tissue invades and destroys the uterine tissue after the manner of a malignant growth. It contains no proper connective tissue stroma, or blood vessels of its own, but seems specially to attack the uterine blood vessels, and its mode of dissemination is by the blood stream.

With the view of clearing up the mode of origin, he gives the result of the latest observations on the chorionic epithelium, on the nature and origin of which and of the "syncytial wandering cells" in the decidua the nature of chorion-epithelioma depends.

He alludes to the views of Langhans, namely, that the inner layer of the chorionic epithelium (layer of Langhans) is of fœtal epiblastic origin, while the syncytium is a proliferation of the uterine epithelium, and shows that these views have been shown

[1] *Monatschr. f. Geburtsh. u. Gynäk.*, Bd. xvii., Hft. 4; abstract in *Brit. Gynæc. J.*, 1903, xix. summary, 139.

[2] *Tr. Obst. Soc. Lond.*, 1903, xlv. 256.

to be incorrect, the work of Siegenbeck van Heukelom and Hubert Peters showing that the whole of the chorionic epithelium is fœtal epiblast, the cells of the layer of Langhans being modified into syncytium when in contact with the maternal blood of the intervillous spaces. Hubrecht in 1889 showed that in the hedgehog the ovum became differentiated into two sets of cells—an outer and an inner. The outer primitive epiblast proliferated rapidly into a thick layer of cells, which became specialised as an organ for the nutrition of the ovum at the expense of the maternal blood, the actual embryonic shield being developed from a part of the outer layers and the inner set of cells together. The thick layer he named the "trophoblast," and around it considerable destruction of the maternal tissues occurred, the degenerated remains being probably used up by the trophoblast for its own nourishment. This edacious action of the trophoblast ceased, and equilibrium was established after a time. When maternal blood-vessels had been opened, the trophoblast became hollowed out into a sponge-work of cavities containing maternal blood which did not coagulate, the cavities taking the place of vessels and becoming part of the maternal circulation, that is, when a primitive placenta had been established, the maternal endothelium had disappeared and its place was taken by fœtal cells. Later the fœtal mesoblast-bearing vessels entered into combination with the trophoblast, and villi were formed, consisting of cores of fœtal mesoblast covered by trophoblast, which then became the epithelium of the villi, and the cavities in it the intervillous spaces.

The ovum in Peter's case shows that similar processes take place in the human ovum, although differences in detail are noted.

The net results of the observations in Teacher's view show that the syncytium develops out of the individual cells of the trophoblast, which are obviously epiblastic in origin, and that so far from the maternal tissues showing proliferation when in contact with syncytium, on the contrary they show degeneration. So that, on Teacher's showing, we are again reminded of the edacious powers of the trophoblast. We are shown that the syncytium is developed from the trophoblast, and that at a certain point in the development of the ovum the edacious power of the trophoblast, neutralised by the resisting power of the maternal tissues, ceases, and equilibrium is established. Hence it is apparent that the trophoblast or epiblastic structure manifests up to a certain point in the development of the ovum many of the characteristics of malignant growth, and these characteristics, if for any reason perverted or stimulated to such a degree that they can overpower the resistance of the maternal tissues, lead to the actual development of diseased conditions, of which chorion-epithelioma is one.

The diseased conditions are: (1) Simple hydatid mole, (2)

malignant hydatid mole, (3) tumours composed almost entirely of syncytium and individual cells, in which a few villi, either normal as to their mesoblastic cores or hydatidiform, are seen; (4) pure chorion-epitheliomata with no trace of fœtal mæsoblast.

Instances of each of these four conditions have been met with and described, and those of the third category, in which all the components of the villi are present, seem conclusive, since in these the whole of the tumour tissue in all its varied cell forms can be traced directly to its source.

Space will not admit of a detailed account of the remainder of Teacher's paper. He concludes as follows:—(1) The so-called deciduoma malignum is a tumour arising in connection with a pregnancy, and originates from the epithelium of the chorionic villi or its forerunner, the trophoblast, which is of ectoblastic origin. (2) That chorion-epithelioma and malignant hydatidiform mole form a characteristic group of tumours clinically and pathologically, and should be classified as neither sarcomata nor carcinomata, but as a distinct class. (3) That in addition to the common tumours developing from a pregnancy there are tumours containing precisely similar structures which are not connected with pregnancy, and may occur in other parts of the body than the uterus and in either sex. These are probably to be explained as being teratomata, originating from some structure which has the morphological value of an included matured and fertilised ovum, and the chorion-epitheliomatous tissue represents the actual trophoblast of the included ovum. The results are as follows:—In 188 cases death occurred in 121 and recovery in 65. In 99 cases out of 188 in which radical operation was performed death occurred in 36 and recovery in 63.

At the same meeting specimens were shown and cases described to the number of 19, all of which occurred in the practice of British observers, a very considerable advance in the amount of material available on that of 1896, when only three cases appear to have been recorded in this country. A prolonged discussion followed, from which we gather that for the most part the speakers agreed with Teacher. A few seemed inclined to hold to the views of Veit as to the sarcomatous type and maternal origin of the characteristic cells.

The previously-noted connection with hydatidiform mole is again emphasised in this paper. Out of the 188 cases recorded 73 occurred in connection with this, 59 in connection with abortion, 49 with full-time pregnancy, and 7 with tubal or ovarian pregnancy. Of the 89 cases not submitted to radical operation 85 died, but, a noticeable fact, 8 patients in whom signs of pulmonary metastasis had occurred all recovered.

The practical and clinical deductions are unaltered. This form of tumour is as a rule highly malignant, and should be dealt with radically as early as possible. To this end all specimens of so-called "retained products of conception" should be submitted to careful microscopical examination, especially if irregular hemorrhages occur after termination of a pregnancy, whether normal or molar, since there is little doubt that in many cases so described and in which death occurred the cause has been in reality chorion-epithelioma malignum, and not the simple one of sepsis or hemorrhage.

WALTER C. SWAYNE.

### Reviews of Books.

